# Prevalence of Osteochondral Lesions on Magnetic Resonance Imaging Following Simple Elbow Dislocations †

**DOI:** 10.3390/jcm14020575

**Published:** 2025-01-17

**Authors:** Jennifer Bruttel, Stephan Regenbogen, Verena Wagner, Heidi Leifeld, Paul A. Grützner, Marc Schnetzke, Philip-Christian Nolte

**Affiliations:** 1 Department for Trauma and Orthopaedic Surgery, BG Klinik Ludwigshafen, Ludwig-Guttmann-Strasse 13, 67071 Ludwigshafen, Germany; jennifer.bruttel@bgu-ludwigshafen.de (J.B.); s_regenbogen@web.de (S.R.); heidi.leifeld@bgu-ludwigshafen.de (H.L.); paul.gruetzner@bgu-ludwigshafen.de (P.A.G.); 2 Medical Faculty, Heidelberg University, Grabengasse 1, 69117 Heidelberg, Germany; verena.steinle@med.uni-heidelberg.de; 3 Clinic for Diagnostic and Interventional Radiology, University Hospital Heidelberg, Im Neuenheimer Feld 420, 69120 Heidelberg, Germany; 4 German Joint Centre, ATOS Clinic Heidelberg, Bismarckstraße 9, 69115 Heidelberg, Germany; marc.schnetzke@atos.de

**Keywords:** simple elbow dislocation, osteochondral lesion, inter-rater reliability

## Abstract

**Objective:** Literature regarding osteochondral lesions in patients following elbow dislocation is scarce. The aim of this study was to examine osteochondral lesions on MRI in patients following simple elbow dislocations and evaluate inter-rater reliability between radiologists and orthopedic surgeons at different levels of experience. **Methods:** In this retrospective, single-center study, 72 MRIs of patients following simple elbow dislocations were evaluated. Ligamentous and osteochondral injuries were evaluated by a junior and senior radiologist and a junior and senior orthopedic surgeon. Osteochondral lesions were classified according to the Anderson classification, and their distribution was assessed. Inter-rater reliability was assessed using Cohen’s Kappa (95% CI) and Fleiss’ Kappa (95% CI). **Results:** The mean time from injury to MRI was 6.92 ± 4.3 days, and the mean patient age was 42.4 ± 16.0 years. A total of 84.5% of patients had a lateral collateral ligament tear, and 69.0% had a medial collateral ligament tear. Osteochondral lesions were found in 27.8% to 63.9% of cases. According to the senior orthopedic surgeon, 100% were first-grade lesions, whereas the senior radiologist classified 63.2% as first-grade, 26.3% as second-grade, and 5.3% as third- and fourth-grade lesions. Inter-rater reliability was fair to moderate for ligamentous injuries and fair for osteochondral lesions (Fleiss Kappa 0.25 [0.15–0.34]). Localization of the lesions differed depending on the examiner. For all examiners, osteochondral lesions of the lateral column (radial head and capitulum) were most common, with 57.8–66.7% of all lesions. Inter-rater reliability was moderate for lesions in the medial column (Fleiss Kappa 0.51 [0.41–0.6]) and fair for lesions in the lateral column (Fleiss Kappa 0.34 [0.24–0.43]). **Conclusions:** Osteochondral lesions following simple elbow dislocations are common; however, in contrast to the current literature, high-grade lesions seem to be relatively rare. Overall inter-rater reliability between radiologists and surgeons, as well as within surgeons, was only moderate to fair regarding ligament and osteochondral lesions.

## 1. Introduction

Elbow dislocations are the second most common dislocated joint, with an incidence of 6 in 100,000 [1,2]. They are divided into simple and complex dislocations. Simple elbow dislocations are classified as a dislocation without accompanying fractures, except fracture of the coronoid tip (type 1 according to Regan and Morrey). The most frequent direction of dislocation is posterolateral, caused by valgus forces with hyperextension [3]. Only about 10% are posteromedial dislocations caused by varus stress and posteromedial rotation [4].

Optimal treatment of simple and complex elbow dislocations is a topic of continuous debate. Outcomes in general are satisfactory if not good [5,6,7,8]. Only very few studies mention or focus on injury patterns after elbow dislocation. Tears of the medial collateral ligament (MCL) are described in 40% to 92% of patients, and tears of the lateral collateral ligament (LCL) in 53% to 97%. Injuries of the common extensor origin (CEO) are described in 6% to 80% and injury of the common flexor origin (CFO) in 12% to 92% [9,10,11,12]. Prevalence of soft tissue injuries varies widely. Most of the studies investigated small patient populations (*n* = 12–20) [9,10,11], and only one of the studies included 147 patients [12].

A myriad of literature regarding elbow dislocation focuses on the treatment and outcome after such injury. Magnetic resonance imaging (MRI) is not included in many routine diagnostic algorithms, as standard radiographs and clinical examination for elbow stability remain the gold standard in decision making in most studies. There is an ongoing debate about the role of MRI in the diagnostic algorithm of elbow dislocations. A cadaveric study found a sensitivity of 88% and a specificity of only 45–75% for full thickness LCL tears and a sensitivity of 55–88% and a specificity of 80–100% for MCL tears [13]. Inter-rater reliability regarding MRI analysis of the elbow differs from poor to good and therefore partly questions the use of MRI findings in clinical decision making [10,13,14,15,16]. When assessing osteochondral lesions, these findings are not limited to the elbow joint. For patients with osteochondritis dissecans of the elbow, inter-rater reliability was found to be only poor to fair [17].

Osteochondral lesions of the elbow in general are typically caused by repetitive trauma, particularly in athletes engaged in throwing sports, or by acute injury [18,19,20]. The most common site for these lesions is the capitulum [21]. Diagnosis usually involves imaging techniques such as plain radiographs, MRI, or arthroscopy. Plain radiographs can only detect advanced lesions, where subchondral sclerosis or displaced fragments may be visible [19,22]. MRI, on the other hand, is preferred for evaluating the size, location, and stability of the lesion and can identify early stage lesions. Osteochondral lesions of the capitulum are most often found in the anterior portion of the capitulum [19,22,23]. Lesions of the trochlea are less frequent and typically occur in athletes subjected to hyperextension forces [24]. A radiological study notes that lesions in the trochlea differ in appearance based on their location. Medial lesions tend to be smaller, usually located on the posterior articular surface, and are associated with high rates of chondral fissuring, subchondral osseous pitting, and marrow edema-like signal changes. In contrast, lesions on the lateral side tend to be larger, more circumscribed, and located on the posteroinferior surface, often resembling classic osteochondritis dissecans [25].

Common complications of osteochondral lesions of the elbow include chronic pain, reduced range of motion, and the eventual development of osteoarthritis [26]. As the lesion advances, patients may experience progressive loss of motion, particularly in elbow extension or flexion, often due to pain, mechanical blockages (such as loose bodies), or the formation of scar tissue around the damaged area [27].

There are a few studies discussing the prevalence of ligamentous injuries after elbow dislocation, but so far only one recent study regarding osteochondral lesions. In this study, MRI analysis was performed on 43 patients following simple elbow dislocations. In almost half of the patients (48.8%), osteochondral lesions were found. The majority of these lesions (69.8%) were first-grade; however, third- and fourth-grade lesions were also found at the capitulum [28].

Few other studies describe osteochondral lesions or bone bruises in patients with simple elbow dislocation. Eygendaal et al. found osteochondral lesions on the capitulum in 20% of their patients, and Schreiber et al. found osteochondral lesions on the capitulum in 18.8% of their patients [2,29]. Demino et al. described a bone bruise in 66.7% of their patients, mostly located at the capitulum (33.3%) and the radial head (41.7%) [9].

These findings suggest that osteochondral lesions may be common in patients following simple elbow dislocations, potentially influencing patient outcomes. However, the literature on the prevalence and distribution of those lesions is limited.

The hypothesis of this study was that low-grade osteochondral lesions are common in patients after simple elbow dislocations but high-grade lesions are scarce. We expected moderate to good inter-rater reliability between the senior examiners, while it might be lower between junior examiners.

The aim of this study was to examine the prevalence of osteochondral lesions on MRI in patients following simple elbow dislocations and their distribution about the elbow joint. Since inter-rater reliability regarding assessment of MRI of the elbow and osteochondral lesions in general ranges from poor to good in the current literature, we also included the evaluation of inter-rater reliability between radiologists and orthopedic surgeons at different levels of experience.

## 2. Methods

In this retrospective, single-center study, MRIs of all patients following simple elbow dislocations treated at a level I trauma center between 2012 and 2021 were evaluated. The study was approved by the local ethics committee. Exclusion criteria were a patient age of less than 16 years, insufficient MRI quality and/or incomplete data, any fracture (except coronoid tip avulsion [Regan Morrey type I]), previous surgery or arthritis of the injured elbow, and time from injury to MRI of more than 21 days.

All included patients underwent minimum 1.5 T MRIs with specific elbow surface coils. Coronal, axial and sagittal images with non-fat-saturated T1-weighted and proton density-weighted sequences, as well as short tau inversion recovery (STIR) sequences or fat-saturated T2/proton density-weighted, were available in all patients.

The MRIs were then individually evaluated by a senior (SR) and junior radiologist (JR), as well as a senior (SOS) and junior orthopedic surgeon (JOS). Injuries to the medial (MCL) or lateral (LCL) collateral ligaments or common extensor (CEO) or flexor (CFO) origins were assessed by a JR, JOS and SOS. Osteochondral lesions were assessed additionally by an SR. Osteochondral lesions were classified according to the Anderson classification, and localizations of the lesions were noted (Table 1) [30]. Localization of the lesions was additionally divided into medial and lateral columns. The medial column included lesions to the trochlea and coronoid tip. The lateral column included lesions to the capitulum and the radial head. More than one lesion per elbow could be noted.

### Statistical Analysis

Continuous data are presented as mean (range) or mean (standard deviation). Categorical values are presented as percentages. Inter-rater reliability between two examiners was assessed using Cohen’s Kappa. To assess inter-rater reliability between all examiners, Fleiss Kappa was used. Statistical analysis was performed with DATAtab statistics calculator (DATAtab e.U., Graz, Austria). To interpret the inter-rater reliability, the recommendation of Landis and Koch was used [31].

## 3. Results

A total of 155 MRIs of patients who had sustained an elbow dislocation between 2012 and 2021 were available. Of those, 83 patients were excluded, leaving 72 patients for further analysis.

The mean time from injury to MRI was 6.92 ± 4.3 days, and the mean patient age was 42.4 ± 16.0 years (range 17–72 years). There were 47.2% female and 52.8% male patients. The right elbow was injured in 46.5% and the left elbow in 53.5% of cases.

### 3.1. Soft-Tissue Injuries

A LCL tear was found in 60.6% of patients by the JOS, in 84.5% by the SOS, and in 66.2% by the JR. MCL injuries were found in 78.9% of patients by the JOS, in 69.0% by the SOS, and in 74.6% by the JR. Injuries of the CEO were described in 39.4% of patients by the JOS, in 50.7% by the SOS, and in 25.4% by the JR, and injuries to the CFO were described in 46.5% of patients by the JOS, in 52.1% by the SOS, and 50.7% by the JR. Avulsion of the coronoid tip was found in 36.6% of patients by the JOS, 26.8% by the SOS, and 42.3% by the JR. Table 2 provides an overview of the soft tissue injuries assessed by the different examiners.

**Figure 1:**
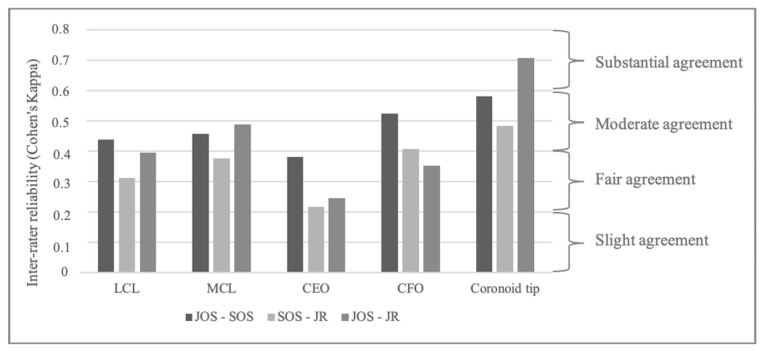
Inter-rater reliability according to Cohen’s Kappa for each of the soft-tissue injuries. CEO: common extensor origin, CFO: common flexor origin, LCL: lateral collateral ligament, MCL: medial collateral ligament. JOS: junior orthopedic surgeon, JR: junior radiologist, SOS: senior orthopedic surgeon.

Inter-rater reliability was fair for injuries of the LCL and CEO (Fleiss Kappa 0.35 [0.22–0.48] and 0.26 [0.13–0.39]) and moderate for injuries of the MCL, CFO and coronoid tip avulsions (Fleiss Kappa 0.41 [0.28–0.54], 0.43 [0.3–0.56] and 0.58 [0.45–0.71]) between all examiners. Substantial agreement between raters was only found between the junior radiologist and the junior orthopedic surgeon for coronoid tip fractures (Cohen’s Kappa 0.71 [0.54–0.87]; Figure 1).

### 3.2. Osteochondral Lesions

Data regarding the osteochondral lesions are summarized in Table 3. Osteochondral lesions were found in 27.8% (JR) to 63.9% (SR) of cases. Overall, first- and second-grade lesions were most common for all examiners. According to the SOS, all lesions (100%) were first-grade lesions, whereas the SR classified 63.2% as first-grade, 26.3% as second-grade, and 5.3% as third- and fourth-grade lesions each. In contrast, the JR classified 75.0% as first-grade, 10.0% as second-grade, 10.0% as third-grade, and 5.0% as fourth-grade lesions. The JOS classified 43.3% as first-grade, 46.7% as second-grade, 6.7% as third-grade, and 3.3% as fourth-grade lesions.

Overall, inter-rater reliability for osteochondral lesions was fair for all examiners (Fleiss Kappa 0.25 [0.15–0.34]). Even more severe osteochondral lesions (third- and fourth-grade lesions) demonstrated only fair agreement (Fleiss Kappa 0.28 [0.18–0.37]).

Agreement on osteochondral lesions was best between the JOS and SOS with moderate inter-rater reliability (Cohen’s Kappa 0.42 [0.25–0.60]) and worst between JR and SR (Cohen’s Kappa 0.06 [−0.1–0.22]).

**Figure 2:**
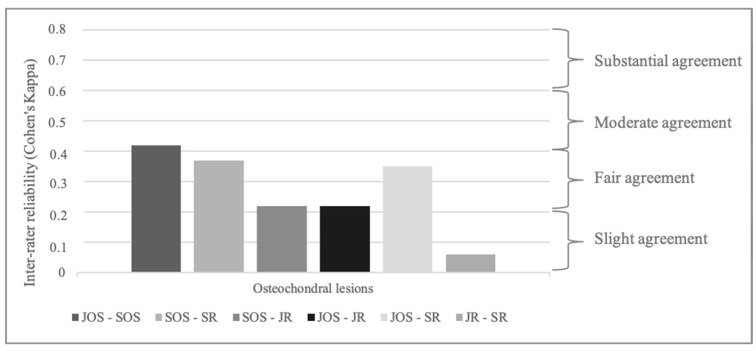
Inter-rater reliability for osteochondral lesion (Cohen’s Kappa). JOS: junior orthopedic surgeon, JR: junior radiologist, SOS: senior orthopedic surgeon, SR: senior radiologist.

Inter-rater reliability for osteochondral lesions was fair between JR and SOS (0.22 [0.07–0.38]), JR and JOS (0.22 [0.03–0.48]), SR and JOS (0.35 [0.17–0.53]), and SR and SOS (0.37 [0.26–0.48]) (Figure 2).

### 3.3. Location of Osteochondral Lesions

Location of the lesions differed depending on the examiner (Table 4). Except for the SR—who found most lesions at the capitulum (45.5%)—all other examiners found most osteochondral lesions at the radial head. Of note, Osborne-Cotterill lesions were found in 9.7–44.4% of patients and were not defined as osteochondral lesions.

For all examiners, osteochondral lesions of the lateral column of the elbow joint (radial head and capitulum) were most common, with 57.8–66.7% (JOS 66.7%, SOS 57.8%, JR 60.0% and SR 65.2%) of all lesions in 16.7–41.7% of all patients.

Osteochondral lesions of the medial column of the elbow joint (trochlea and coronoid) were observed in 17.4–40.0% (JOS 22.2%, SOS 20.0%, JR 40.0% and SR 17.4%) of all lesions in 8.3–12.5% of all patients. A total of 0–22.2% (JOS 11.1%, SOS 22.2%, JR 0% and SR 17.4%) of osteochondral lesions were located on both columns in 0.0–13.9% of all patients.

**Figure 3:**
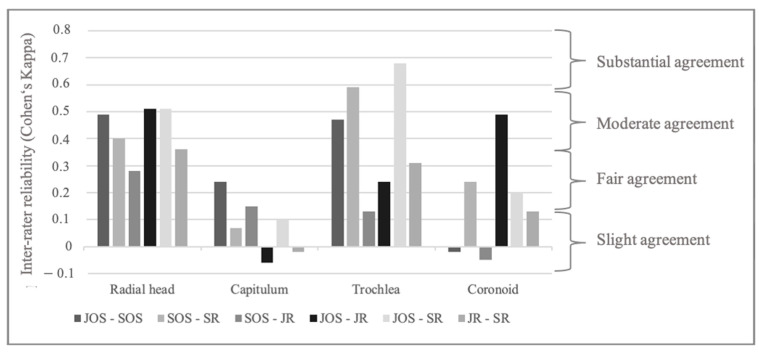
Inter-rater reliability for each location of osteochondral lesion (Cohen’s Kappa). JOS: junior orthopedic surgeon, JR: junior radiologist, SOS: senior orthopedic surgeon, SR: senior radiologist.

Inter-rater reliability for location of the lesion was best for the radial head (Fleiss Kappa 0.4 [0.31–0.5]) and trochlea (Fleiss Kappa 0.41 [0.31–0.51]) with moderate agreement. Agreement was worst for lesions on the capitulum with slight inter-rater reliability (Fleiss Kappa 0.02 [−0.08–0.11]). Figure 3 gives an overview of inter-rater reliability for lesion location between the different examiners.

Inter-rater reliability was slight for Osborne-Cotterill lesions (Fleiss Kappa 0.17 [0.08–0.27]).

**Figure 4:**
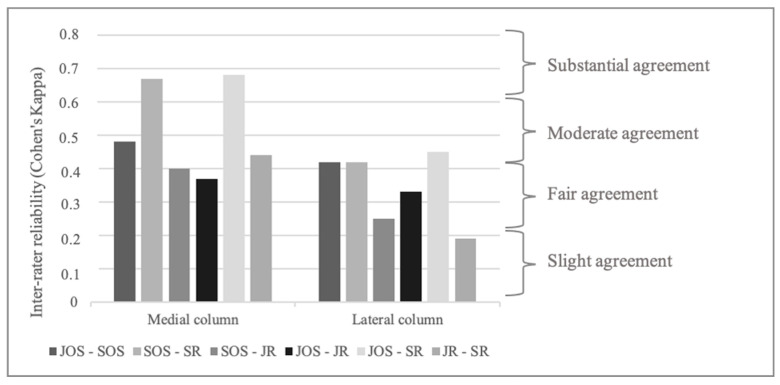
Inter-rater reliability for osteochondral lesions in the medial and lateral column of the elbow. JOS: junior orthopedic surgeon, JR: junior radiologist, SOS: senior orthopedic surgeon, SR: senior radiologist.

Inter-rater reliability was moderate for lesions in the medial column (Fleiss Kappa 0.51 [0.41–0.6]), fair for lesions in the lateral column (Fleiss Kappa 0.34 [0.24–0.43]) and poor for lesions in both columns (Fleiss Kappa 0.2 [0.1–0.29]). Substantial agreement was found between JOS and SR (Cohen’s Kappa 0.68 [0.38–0.98]) and SOS and SR (Cohen’s Kappa 0.67 [0.38–0.95]) for lesions in the medial column (Figure 4).

## 4. Discussion

One of the most important findings of our study was that when interpreting MRIs for osteochondral lesions following simple elbow dislocations, caution is required since there is only poor to moderate inter-rater reliability, with their prevalence ranging from 27.8% to 63.9% depending on the examiner. Furthermore, there seems to be no difference in the agreement of senior and junior examiners or radiologists and orthopedic surgeons.

In this study, injuries to the medial and lateral collateral ligaments were found in 69.0–78.9% and 60.6–84.5% of patients, respectively. When compared to the limited data that are available, our findings are within the range of 40–92% (MCL) and 53–97% (LCL) that has been reported previously [9,10,11,12]. Most of these studies investigated small study populations (*n* = 12–20) [9,10,11], with only a few exceptions [12], making the present study one of the largest.

Comparing our results regarding osteochondral lesions with the above-mentioned study by Kim et al., results do not differ greatly [28]. In their study, MRIs were evaluated by a senior radiologist and two senior orthopedic surgeons, and a consensus was found with the help of a third senior orthopedic surgeon if there was a disagreement. Inter-rater reliability was not reported. Overall, these findings corroborate our results. Especially regarding the column of the elbow joint, their findings are quite similar, as Kim et al. have found most osteochondral lesions in the lateral column of the joint (69.8% vs. 57.8–66.7%) [28]. Furthermore, first-grade lesions were most common in our study as well. However, we did not find too many high-grade lesions in general or at the capitulum as was described by Kim et al. [28].

To this point, clinical significance of especially lower grade osteochondral lesions is not known. If one is looking at osteochondral lesions of the elbow in general, high-grade lesions are generally treated surgically to improve outcome [32,33,34]. Low-grade lesions are usually treated conservatively, which includes an initial resting period and physical therapy afterwards [34,35]. Most patients with simple elbow dislocation undergo conservative treatment, which is quite similar [5,36]. Higher grade osteochondral lesions seem to be rare, but these are the cases which might profit from changing to a surgical treatment plan.

Most elbow dislocations are posterolateral, where valgus forces place stress on the radial head and capitulum [37]. As such, osteochondral lesions are typically expected in the lateral column. In our study, all examiners identified osteochondral lesions in the radial column as the most common, with prevalence rates ranging from 16.7% to 41.7% of all patients (or 57.8% to 66.7% of all lesions), depending on the examiner. These findings align with the predominance of posterolateral dislocations. In contrast, osteochondral lesions in the medial column were less common, affecting only 8.3% to 12.5% of patients. However, detailed data on dislocation direction were unavailable for most patients, as many had already undergone reduction by the time they presented at our clinic.

In the literature, inter-rater reliability regarding MRI analysis of the elbow differs from poor to good. In 2001, two cadaveric model studies by Carrino et al. showed good inter-rater reliability between two radiologists for MCL tears but only poor to moderate inter-rater reliability for LUCL tears depending on MRI sequence [13,15]. Interestingly, we have found agreement to be higher for MCL injuries compared to LCL injuries as well.

In a study by Schnetzke et al., four examiners (2 radiologist and 2 orthopedic surgeons) assessed 30 MRIs of elbows and demonstrated fair to moderate agreement for collateral ligament injuries (Cohen’s Kappa 0.275–0.441) and poor agreement for extensor and flexor injuries (Cohen’s Kappa 0.049–0.413) [16].

On the other hand, inter-rater reliability for soft tissue injury pattern in simple elbow dislocations was found to be substantial (Cohen’s Kappa 0.70) between two radiologists by Luokkala et al. in 17 patients [10]. Bowman et al. found similar inter-rater reliability for full thickness MCL tears in 45 patients between three orthopedic surgeons (Cohen’s Kappa 0.63) [14].

Overall, inter-rater reliability regarding ligamentous injuries of the elbow after simple elbow dislocation in the literature is comparable and sometimes higher than what we have found in our investigation.

In contrast, one retrospective study comparing MRI results to intraoperative findings found a 100% sensitivity and 89% specificity for full-thickness MCL tears and a 79% sensitivity and 100% specificity for full-thickness LCL tears. MR-Arthrography was even found to have 100% sensitivity and specificity for both [38]. When looking at these results, a potential bias must be considered, because it was a retrospective study and known that all patients had undergone surgery for ligamentous injuries.

Regarding osteochondral lesions in 20 osteochondrosis dissecans patients, inter-rater reliability for overall 33 examiners (18 orthopedic surgeons and 15 radiologists) showed only poor to fair agreement (Fleiss Kappa 0.12–0.23). They found no difference in agreement regarding specialty or experience of the examiner [17]. This aligns with the findings of our study where no difference in agreement could be found regarding specialty or experience and only fair inter-rater reliability for osteochondral lesions.

Overall, the results of this study regarding inter-rater reliability show that agreement between radiologists and orthopedic surgeons on all levels of experience is only poor to moderate. This leads to the assumption that assessing osteochondral lesions in MRIs of the elbow can be challenging. There are some pitfalls like pseudodefects on the posterior surface of the capitulum and the midtrochlea notch which are physiologically without cartilage and can therefore be mistaken for osteochondral lesions [39,40]. Additionally, since this was a retrospective study including imaging from 2012 to 2021, most MRIs were 1.5 tesla or less and quality of imaging varied vastly. With the results of this study in mind, the imaging results after elbow dislocations should be discussed between the radiologist and orthopedic surgeon before a treatment decision is made and ideally the MRI should be assessed together. Additionally, including clinical findings and further imaging modalities such as MR arthrography in the diagnostic algorithm should be considered.

The inclusion of MRI in the standard diagnostic workflow for elbow dislocations remains a topic of ongoing debate, which may contribute to its underuse in these patients. Based on the findings of this study and the existing literature, the limited inter-rater reliability of MRI makes it important to approach MRI findings in elbow dislocation cases with caution. As a result, incorporating MRI as a routine part of the decision-making process for treatment currently does not appear to be justified. However, with advancements in MRI technology, particularly the increased use of 3 Tesla MRIs, this approach may evolve in the near future. To address this uncertainty, further studies utilizing higher-resolution MRI techniques, along with research comparing MRI findings to intraoperative findings, are necessary.

In general, the significance of osteochondral lesions following simple elbow dislocations is limited, as most patients achieve good outcomes after nonoperative treatment, despite many of them having osteochondral lesions of some degree. However, there are some patients with unsatisfactory outcomes and further research should investigate if osteochondral lesions play a role. One of the main complications of simple elbow dislocations is stiffness of the joint, which can also be a consequence of osteochondral lesions [26,27]. A correlation of osteochondral lesions in imaging and clinical outcome should therefore also be a matter of future research.

### Limitations

This study has various limitations. One of them is that although we had minimal technical requirements, the quality of MRIs was still heterogeneous. The diagnostic algorithm used for evaluating the imaging in this study was somewhat vague. A more detailed and systematic approach to MRI assessment could have potentially improved inter-rater agreement among the examiners. With 3T MRIs becoming more available, detection and agreement of osteochondral lesions may become easier. Additionally, to better compare our results to the body of literature, a consensus result between the examiners would have been helpful. A comparison of radiological results to intraoperative findings would have facilitated verifying MR findings; however, the gold standard for simple elbow dislocations remains nonoperative treatment and most patients do not undergo surgery [5,36]. Another limitation is the use of a single imaging modality for the diagnosis. Including, for example, MR arthrography could have improved diagnostic accuracy.

## 5. Conclusions

Osteochondral lesions were found to be common in this study, although higher-grade lesions appeared to be less frequent. However, it is crucial to interpret these and other MRI findings regarding osteochondral lesions of the elbow with caution. This study, in alignment with the available literature, has demonstrated poor to fair inter-rater reliability for MRI assessment of osteochondral lesions, even among experienced examiners. Furthermore, research specifically on osteochondral lesions following elbow dislocations remains limited. This study highlights the need for further investigation. Future research should prioritize higher-resolution MRI imaging and compare MRI findings with intraoperative findings. Additionally, more detailed diagnostic algorithms for MRI assessment of osteochondral lesions of the elbow are necessary to improve inter-rater agreement and enhance diagnostic accuracy.

## Figures and Tables

**Table 1:** Anderson classification of osteochondral lesions [30].

Grade	Definition
1	Subchondral edema
2	Partially detached osteochondral lesion with edema
3	Fully detached, non-displaced osteochondral lesion
4	Displaced osteochondral fragment

**Table 2:** Prevalence of soft-tissue injuries for the different examiners in % of all patients. JOS: junior orthopedic surgeon, JR: junior radiologist, SOS: senior orthopedic surgeon.

Injury	JOS	SOS	JR
Lateral collateral ligament	60.6%	84.5%	66.2%
Medial collateral ligament	78.9%	69.0%	74.6%
Common extensor origin	39.4%	50.7%	25.4%
Common flexor origin	46.5%	52.1%	50.7%
Coronoid tip	36.6%	26.8%	42.3%

**Table 3:** Grade of osteochondral lesion according to the Anderson classification of all lesions. JOS: junior orthopedic surgeon, JR: junior radiologist, SOS: senior orthopedic surgeon, SR: senior radiologist.

Grade	JOS	SOS	JR	SR
1	43.3%	100%	75.0%	63.2%
2	46.7%	0%	10.0%	26.3%
3	6.7%	0%	10.0%	5.3%
4	3.3%	0%	5.0%	5.3%

**Table 4:** Location of osteochondral lesions in % of all lesions. JOS: junior orthopedic surgeon, JR: junior radiologist, SOS: senior orthopedic surgeon, SR: senior radiologist.

Location	JOS	SOS	JR	SR
Radial head	51.4%	54.1%	39.1%	25.8%
Capitulum	20.0%	13.1%	13.0%	45.5%
Trochlea	25.7%	24.6%	34.8%	18.2%
Coronoid	2.9%	8.25%	13.0%	10.6%

## Data Availability

All data and materials regarding the study are available from the corresponding author.
